# Before we tell people to avoid ultra-processed foods, can they identify them? The case for cognitive validity criteria in food classification

**DOI:** 10.1017/S1368980026103036

**Published:** 2026-07-09

**Authors:** Jimmy Chun Yu Louie

**Affiliations:** Discipline of Dietetics, Swinburne University of Technology, Australia

**Keywords:** Ultra-processed foods, Nova classification, Lancet series, Dietary policy, Regulatory fitness, Classification validity, Evidence appraisal

## A policy in search of a user

In little more than a decade, the idea that industrial food processing carries health risks beyond nutrient content has moved from research hypothesis to a fixture of national dietary guidance^(1,2)^. Brazil^(3)^, Canada^(4,5)^ and others now advise their populations to limit ultra-processed foods (UPF)^(6,7)^, and mandatory front-of-pack identification is under discussion in several jurisdictions^(8)^. The advice rests on consistent cohort evidence linking high Nova Group 4 intake to obesity, CVD, cognitive health, type 2 diabetes and premature mortality^(9–11)^.

One question has gone curiously unattended. The guidance asks people to act on a category, yet we have little assurance they can recognise it in the supermarket. The scholarly debate over Nova has centred on whether the classification is scientifically sound: whether processing predicts health independently of nutrients, whether coders apply it reliably and whether its boundaries hold^(12–14)^. Those questions remain unsettled, but they are not the one a shopper faces, which is simpler: *which products am I being told to avoid?* This commentary argues that the question belongs at the centre of nutrition policy, not its periphery.

## What happens when people try to apply Nova

The available evidence is not encouraging. Surveys across multiple countries suggest that consumers have limited and often inaccurate understanding of the Nova classification system and the concept of UPF, frequently interpreting it in terms of nutrient content (e.g. sugar, fat or additives) rather than the industrial formulation criteria that Nova uses^(15,16)^. A recent study in the UK indicates substantial confusion around category boundaries, particularly between processed and UPF, with many individuals struggling to distinguish between these groups in practice^(17)^. Errors tend to occur in a consistent direction: foods perceived as nutritious, such as wholegrain products or yogurt, are often judged to be less processed regardless of their additive content, reflecting reliance on healthfulness cues rather than formal classification rules^(16)^.

Qualitative research across diverse settings reinforces this pattern, showing that people hold coherent and confident beliefs about food processing, but that these beliefs are shaped by visual appearance, perceived naturalness and familiar nutritional heuristics rather than Nova’s criteria^(17,18)^.

It would be easy to read this as low nutrition literacy, with better education the remedy. That reading is too quick. People are not failing to think about processing; they think about it carefully, using rules that happen not to match Nova’s^(19,20)^. Why those rules persist is why education has limited prospects^(21,22)^.

## Three reasons the problem is built into the instrument

Nova was built for a legitimate purpose: to code foods in dietary surveys so researchers could study processing as a population exposure^(1)^. For that, it performs well. The difficulty arises when the same instrument must guide individual purchases, where three features become decisive obstacles.

The first is what Nova measures. Group 4 membership turns on additives serving cosmetic or functional roles, such as emulsifiers, modified starches and artificial flavours^(1)^. These are invisible on inspection, and recognising them requires additive fluency few consumers possess. Asked to reason aloud through ingredient lists, adults routinely mistook emulsifiers and stabilisers for natural components^(23,24)^. Qualitative and observational research shows that ingredient lists are frequently experienced as opaque, with unfamiliar chemical names and codes discouraging meaningful interpretation and leading consumers to rely on heuristics instead^(25,26)^. The defining criterion is, for most people, illegible^(25,26)^.

The second is where the key boundary sits. The distinction guidance depends on is between Group 3, which it permits, and Group 4, which it targets, yet that line is drawn on an additive’s purpose rather than its amount, a judgement about industrial intent that even experts make by convention^(27)^. Inferring intent from a label in the two seconds shoppers spend on a package is not the kind of analysis the situation permits^(28)^.

The third is consistency across products people meet. Because Group 4 status depends on formulation, the same food shifts between groups from one brand to the next^(14)^: a bread loaf may be Group 3 or 4 depending on an emulsifier, and a yogurt Group 1, 3 or 4 depending on its flavourings^(13,14,27)^. Someone who learns one product’s classification cannot carry it to the next purchase and gets no signal when the inference quietly fails. At the population level, these variations average out; at the individual decision level, where guidance operates, the instability is disabling.

## Why more education is the wrong lever

**Figure 1.**
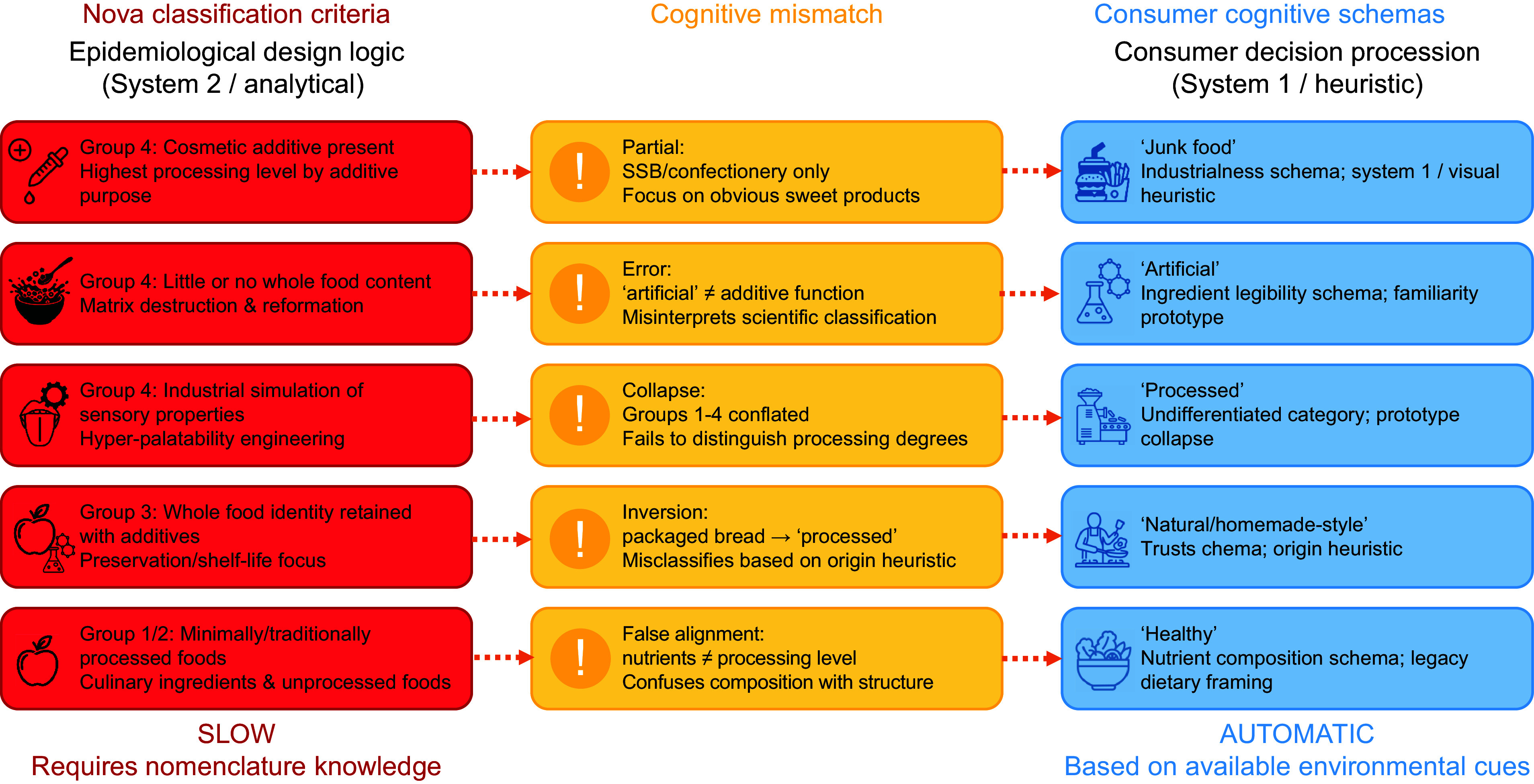
Where Nova classification criteria diverge from the cognitive schemas consumers use to judge food. SSB, sugar-sweetened beverage.

If people simply lacked information, supplying it would help. The deeper obstacle is that they already have well-developed ways of categorising food, doing exactly what cognition should under time pressure. Fast, automatic judgement dominates decisions made with limited attention^(29)^, and food shopping is the textbook case. Three habitual schemas recur in the literature; Figure 1 sets them against the Nova criteria they approximate.

People judge processing by industrial origin, treating factory packaging as the signal and artisanal presentation as its absence^(30)^. They judge it by ingredient legibility, reading familiar names as wholesome and unfamiliar ones as suspect, a strategy that misfires on clean-label reformulations and on traditional foods with long ingredient lists alike^(29)^. Above all, they judge it by nutritional content, the dimension four decades of guidance has trained them to read, so a fortified cereal reads as wholesome and a high-protein processed meat as benign^(19,31)^. Each schema is sensible, and each diverges from Nova in its own predictable way. None is a deficit a leaflet can repair, because the schemas operate beneath awareness and are reinforced by the food environment itself^(29,30)^. Layering Nova over them, without changing what the label shows, leaves the machinery untouched.

## Four cognitive validity criteria

**Figure 2.**
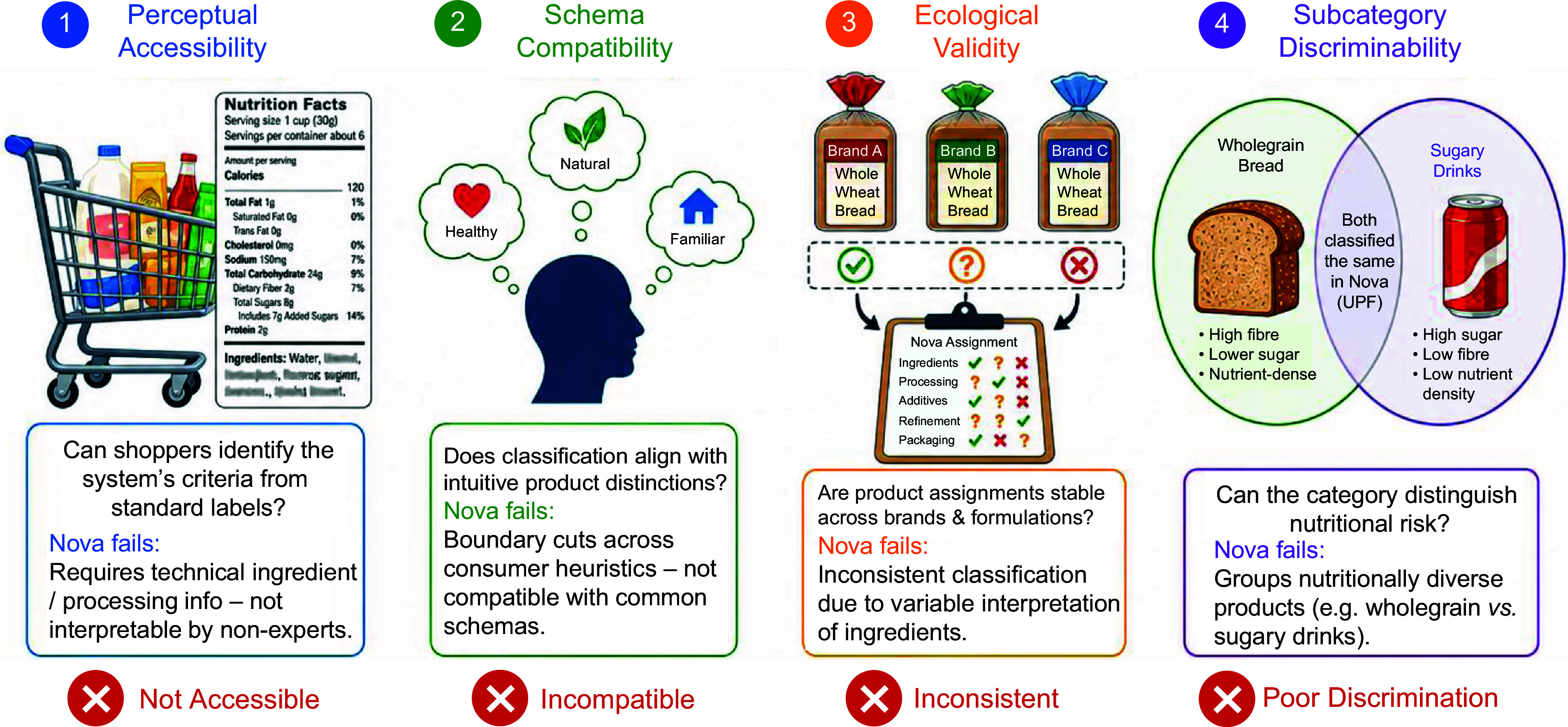
Four key criteria for evaluating food classification system and how Nova fails to achieve all of them.

If a research instrument has been deployed for guidance without testing its fitness, the constructive response is to specify what such a test involves. Nutrition validates dietary assessment and screening tools before fielding them^(32)^; consumer-facing classification deserves the same discipline. I propose four criteria (Figure 2), each tied to a failure above and answerable by a feasible study.

*Perceptual accessibility* asks whether the defining criterion can be identified from information routinely present on the product, without specialist knowledge. It can be tested by asking untrained shoppers to classify products from the label alone and comparing their assignments with those of trained coders. Evidence from food‑labelling research shows that even comparatively simple nutrition information depends critically on readability and comprehension and becomes unreliable when information is opaque or technically framed^(33)^.

*Schema compatibility* asks whether the classification boundary maps onto distinctions people can already draw, or whether it cuts across them. It can be tested by analysing the structure of consumer errors to see whether they are random or systematically inverted. Work on food perception consistently shows that consumers apply stable heuristic schemas based on cues such as naturalness, familiarity and perceived healthiness that structure judgements in systematic rather than random ways^(19,31)^.

*Ecological validity* asks whether the system yields stable assignments across the branded variants of a food that a shopper meets in practice. It can be tested by re‑classifying matched products from the same category across brands and measuring agreement. Evidence on Nova indicates that classification can vary depending on interpretation of ingredients, formulation and context, leading to inconsistent assignment and risk of misclassification across studies^(34,35)^.

*Subcategory discriminability* asks whether a guidance category groups together products of materially different nutritional risk, and if so whether it offers any basis for telling them apart. It can be tested by examining whether the category supports nutritionally sensible substitution or forces shoppers to treat wholegrain bread and sugary drinks as equivalent. A large body of evidence shows substantial nutritional heterogeneity within UPF, with some subgroups (e.g. sugar‑sweetened beverages) consistently associated with harm, while others (e.g. wholegrain cereals and breads) show neutral or even beneficial associations^(9,36)^.

Applied to Nova, the criteria converge. The system fails perceptual accessibility because its criterion is illegible on the label, schema compatibility because its boundary is orthogonal to the schemas people use, ecological validity because formulation varies by brand and subcategory discriminability because Group 4 spans the trivial and the important alike^(13,14,34)^. This is no reason to discard what Nova has taught us about diet and health, but every reason to stop assuming a research variable can serve unmodified as a shopping aid.

## Implications for nutrition policy

Three consequences follow. First, the policy lever is being pulled in the wrong place: resources spent teaching the public to recognise UPF will yield little while the obstacle is structural. The productive levers are reforming labelling so the relevant information becomes visible, or adopting criteria people can apply, such as the nutrient-threshold warning labels that shifted purchasing in Chile and Mexico using cues shoppers can read^(37,38)^.

Second, heterogeneity within Group 4 is an equity issue. Fortified breads, plant-based milks and infant formulas sit alongside confectionery and soft drinks^(14,33)^, yet for households on tight budgets they are affordable sources of fibre, Ca and micronutrients that are hard to replace^(39,40)^. Undifferentiated avoidance advice asks most of those least able to absorb the cost, on the basis of a category they are least equipped to apply^(27,41)^. Guidance that ignores this risks widening the very inequalities^(42)^ public health nutrition exists to narrow.

Third, the cultural reach of Nova guidance deserves scrutiny. The domestic–industrial boundary it encodes reflects one food culture, and many non-Western staples, from commercial tortillas to packaged fermented soya products, fall into Group 4 through additives absent from the products that motivated the category^(14,40)^. More broadly, reviews note that Nova’s classification can fail to account for cultural food traditions, leading to inconsistent categorisation across different dietary contexts and populations^(14)^. Exported without adaptation, the guidance may be culturally misdirected as well as confusing.

## Conclusion

The case for caution about UPF does not depend on consumers being able to classify them, but the case for telling them to avoid these foods certainly does. We are issuing instructions in a vocabulary the audience does not share and then attributing the confusion to a literacy problem on their side rather than a design problem on ours. The four criteria offer a way to test that judgement rather than assert it and to hold any future system to a standard of consumer fitness before deployment. Until a system meets it, public health nutrition would do better to base consumer guidance on cues people can see and apply and to reserve Nova for the research role it was built to perform. Whether people can use the tools we give them is the point at which the science either reaches the public or fails to.
